# Refined Carbohydrate-Rich Diet Promotes Early Myocardial Dysfunction Associated with Adiponectin Reduction, Inflammation, and Impaired Ca^2+^ Handling in Female Rats

**DOI:** 10.3390/nu18142281

**Published:** 2026-07-12

**Authors:** Fernanda Calote, Gabriel Coelho Sandoval, Karoline Neumann, Emanuelle Coutinho de Oliveira, Filipe Martinuzo Filetti, Mirian Fioresi, Andressa Bolsoni-Lopes, Karolini Zuqui Nunes

**Affiliations:** 1 Postgraduate Program in Nutrition and Health, Health Sciences Center, Federal University of Espírito Santo, Vitória 29040-091, ES, Brazil; fernanda.calote@edu.ufes.br (F.C.); andressa.lopes@ufes.br (A.B.-L.); 2 Department of Nursing, Health Sciences Center, Federal University of Espírito Santo, Vitória 29040-091, ES, Brazil; gabriel.sandoval@edu.ufes.br (G.C.S.); emanuelle.c.oliveira@edu.ufes.br (E.C.d.O.); 3 Postgraduate Program in Physiological Sciences, Health Sciences Center, Federal University of Espírito Santo, Vitória 29040-091, ES, Brazil; karoline.gomes@edu.ufes.br; 4 Postgraduate Program in Nursing, Health Sciences Center, Federal University of Espírito Santo, Vitória 29040-091, ES, Brazil; filipemartinuzofiletti@gmail.com (F.M.F.); mirian.fioresi@ufes.br (M.F.)

**Keywords:** refined carbohydrate-rich diet, cardiac contractility, inflammation, excitation-contraction coupling, adiponectin

## Abstract

**Introduction**: Diets rich in refined carbohydrates are associated with cardiometabolic dysfunction, but the mechanisms linking metabolic disturbances to myocardial impairment in females remain unclear. This study investigated the effects of short-term consumption of a refined carbohydrate-rich diet on papillary muscle contractility in female rats. **Methods**: Adult female Wistar rats were assigned to either a control diet (CT) or a high-carbohydrate diet (HCD) for 15 days. The HCD consisted of 45% sweetened condensed milk, 10% refined sugar, and 45% standard chow. Metabolic, inflammatory, functional, and molecular parameters were evaluated. Myocardial contractility and intracellular Ca^2+^ handling were assessed in isolated left ventricular papillary muscles, while phospholamban (PLB), SERCA2a, and NCX1 protein expression were analyzed. **Results**: The HCD increased ovarian adipose tissue, triglyceride levels, glucose intolerance, and TNF-α levels, while decreasing HDL cholesterol and circulating adiponectin. Leptin and MCP-1 levels were unchanged. Functional assessments revealed impaired cardiac contractility, evidenced by reduced papillary muscle force and lower positive force derivative (dF/dt^+^). HCD also decreased post-pause potentiation and post-rest contraction, indicating impaired sarcoplasmic reticulum (SR) Ca^2+^ storage and release, as well as potentially reduced extracellular calcium influx. At the molecular level, PLB expression was increased, whereas SERCA2a and NCX1 expression remained unchanged. **Conclusions**: Short-term consumption of a refined carbohydrate-rich diet induces early cardiac contractile dysfunction in female rats, associated with impaired Ca^2+^ handling, reduced adiponectin, and increased inflammation, suggesting that metabolic and inflammatory disturbances precede structural cardiac alterations and may promote cardiometabolic disease development.

## 1. Introduction

The increasing westernization of dietary habits has promoted a substantial rise in the consumption of ultra-processed foods, which are characterized by high levels of refined carbohydrates, fats, and sodium. This dietary pattern has been consistently associated with a higher incidence and progression of cardiovascular diseases [1,2,3]. This scenario is particularly relevant among young populations, in which the intake of refined carbohydrates frequently exceeds the recommendations of the World Health Organization (WHO), which advises limiting this consumption to less than 10% of total daily energy intake, with 5% considered the ideal threshold for additional health benefits [4].

Carbohydrates can be classified as simple or complex according to their chemical structure. Simple carbohydrates include monosaccharides (e.g., glucose and fructose) and disaccharides (e.g., sucrose), whereas complex carbohydrates consist of longer chains of sugar molecules. Among simple carbohydrates, refined sugars, such as sucrose and other added sugars produced through extensive industrial processing, are particularly noteworthy [5]. These are commonly found in products such as soft drinks, candies, sweets, cakes, cookies, and condensed milk [1]. These foods, typically characterized by a high glycemic index and low fiber content, promote rapid increases in blood glucose and insulin secretion, contributing to metabolic disorders such as insulin resistance, dyslipidemia, and increased visceral adiposity [6,7,8].

In addition, the WHO highlights that excessive consumption of free sugars is associated with a higher risk of weight gain, obesity, type 2 diabetes, and other chronic non-communicable diseases [4]. Growing evidence suggests that this dietary pattern contributes to the development of a chronic low-grade inflammatory state, characterized by elevated levels of pro-inflammatory cytokines and reduced concentrations of mediators with cardioprotective properties [9,10].

Studies conducted in animal models have demonstrated that the consumption of diets rich in refined carbohydrates intensifies inflammatory and oxidative processes, resulting in metabolic dysfunction, including alterations in circulating levels of adiponectin, resistin, and leptin [11,12], which may directly affect the cardiovascular system. Reduced adiponectin levels have been associated with impaired cardiac function, as experimental models have shown that adiponectin deficiency or decreased circulating levels are related to worsening cardiac contractile function [13].

In the study by Neumann et al. (2025), the consumption of a diet rich in refined carbohydrates for fifteen days was sufficient to impair endothelial function, characterized by reduced vascular reactivity and increased oxidative stress [14]. In cardiac tissue, most evidence regarding impaired contractility derives from studies using sucrose as the primary dietary source, predominantly in male animals. These studies have demonstrated alterations in Ca^2+^ homeostasis, including reduced myocyte sensitivity to intracellular Ca^2+^ [15] and depressed sarcoplasmic reticulum (SR) function [16]. The increase in pro-inflammatory mediators, such as TNF-α, in cardiac tissue, as well as alterations in the phospholamban–SERCA2a axis [17]. In this context, the regulation of SERCA2a by phospholamban (PLB) depends on a dynamic balance between its total abundance and its phosphorylation state, which determines its inhibitory capacity on the pump. Thus, although phosphorylation represents the main mechanism for acute modulation of this interaction, changes in total PLB expression may alter the functional PLB/SERCA2a ratio, impairing Ca^2+^ reuptake, especially under conditions of metabolic stress [18].

Although most experimental studies evaluating the effects of metabolic disorders on cardiac function have been conducted in male animals, growing evidence indicates that cardiovascular physiology and pathophysiology present important sex-specific differences. In this context, sex hormones, particularly estrogen, play a relevant role in modulating the cardiovascular response to metabolic stress, exerting cardioprotective effects through various cellular and molecular mechanisms [19].

In addition, growing evidence indicates that estrogen plays a fundamental role in maintaining intracellular Ca^2+^ homeostasis in cardiomyocytes [20,21]. These effects may modify the myocardial response to diets rich in refined carbohydrates and sugars, potentially influencing the development and progression of cardiac dysfunction. However, the lack of studies directly assessing myocardial mechanical function in females, particularly in sensitive experimental preparations such as papillary muscle, limits the understanding of the underlying mechanisms. Therefore, investigation in female animals is essential to broaden the understanding of sex-specific mechanisms involved in diet-induced cardiac alterations and to enhance the translational relevance of experimental findings. Accordingly, the present study aimed to investigate the effects of a diet rich in refined carbohydrates on papillary muscle contractility in female rats.

## 2. Materials and Methods

### 2.1. Drugs and Reagents

Xylazine was obtained from Ceva (Paulínia, São Paulo, Brazil), and ketamine from Syntec (São Paulo, Brazil). Isoproterenol (isoproterenol hydrochloride), caffeine, and dihydroethidium (DHE) were purchased from Sigma-Aldrich (St. Louis, MO, USA). All other salts and reagents, analytical grade, were obtained from Sigma-Aldrich or Merck (Darmstadt, Germany). All drugs were diluted in distilled water.

### 2.2. Animals

Adult female Wistar rats (8 weeks old, weighing approximately 250 g) were housed under controlled environmental conditions (23–25 °C) with a 12 h light–dark cycle and had ad libitum access to filtered water and their respective experimental diets: standard chow (CT group) or a high-carbohydrate diet (HCD group). The HCD consisted of standard chow supplemented with refined table sugar (sucrose) and sweetened condensed milk, a food product with a high content of refined sugar, to mimic a Westernized dietary pattern. This study was approved by the Ethics Committee on Animal Use of the Federal University of Espírito Santo (CEUA-UFES, protocol no. 16/2022) and conducted in accordance with the guidelines of the National Council for the Control of Animal Experimentation (CONCEA).

Animals were randomized into experimental groups based on similar baseline body weight prior to the initiation of treatments. Although formal blinding procedures were not applied, all analyses were conducted under standardized methodological rigor, ensuring uniform handling and minimizing potential bias. Group allocation (CT and HCD) remained known throughout the experiments to ensure proper sample identification and technical control during the procedures.

### 2.3. Experimental Groups

Animals were initially subjected to a two-week acclimatization period and subsequently randomly assigned to two experimental groups: (CT and HCD). Both groups were treated for 15 days.

The CT received a standard diet (Socil^®^ feed), whereas the experimental group HCD was subjected to a diet rich in refined carbohydrates, formulated by mixing 395 g of ground conventional feed, 395 g of commercial sweetened condensed milk (Triângulo^®^,Alimentos Triângulo Mineiro, Canápolis, Minas Gerais, Brazil) composed of pasteurized milk, milk powder, sugar, lactose, and stabilizers: sodium triphosphate, monosodium phosphate, and disodium diphosphate), and 83.7 g of refined sugar (União^®^, Camil Alimentos, São Paulo, Brazil). The diets were prepared using solid ingredients weighed on a weight/weight (*w*/*w*) basis. Water (102 mL) was added during preparation and subsequently removed by drying, resulting in a standardized final dry mass for both diets. Based on the final formulation, approximately 36.7% of the dry diet consisted of carbohydrates derived from refined sugar and sweetened condensed milk. This formulation was adapted from previously published protocols [12,22], which demonstrated that diets containing approximately 30% refined sugars, predominantly sucrose, are effective in inducing metabolic and cardiovascular alterations in experimental models. The energy density (kcal/g) and the percentage distribution of carbohydrates, lipids, and proteins in the experimental diets are presented in Table 1.

A total of 56 animals were included in this study, with 28 rats allocated to the CT group and 28 to the HCD group. Minor variations in the final number of animals analyzed in each assay resulted from predefined exclusion criteria, such as sample loss during processing, adverse events unrelated to the experimental protocol, or inconsistent experimental data.

### 2.4. Glucose and Insulin Tolerance Tests

On the twelfth day of treatment, animals underwent a 6 h fasting period prior to glucose tolerance testing (GTT) and insulin tolerance testing (ITT). For the GTT, glucose was administered intraperitoneally (ip) (2 g/kg body weight; 20% solution; Neon Comercial Reagentes Analíticos LTDA, Suzano, Brazil), and blood samples were collected from the tail at 0, 15, 30, 60, and 90 min after injection. For the ITT, animals received regular human insulin (0.75 mU/g body weight; Humulin^®^, Lilly, São Paulo, Brazil), and blood samples were collected at 0, 3, 6, 9, 12, and 15 min. Glucose concentrations were measured using a glucometer (Accu-Chek Active^®^, Roche, São Paulo, Brazil). Glycemic response was assessed based on glucose level variation over time, and results were analyzed by comparing group means [23].

### 2.5. Body Parameters, Tissue Collection, and Glycemic Profile

Body weight gain was calculated as the difference between final and initial body weight (g) throughout the experimental period. Daily food and water intake were determined as the difference between the amount offered and the remaining amount and expressed as g/animal/day. Feed efficiency was calculated as the ratio between body mass gain (g) and energy intake (kcal).

After 15 days of treatment, animals were fasted for 8 h, and fasting blood glucose was measured before anesthesia. Blood samples from the tail artery were used for glucose determination with a glucometer (Accu-Chek Active^®^, Roche, Brazil). Animals were then anesthetized with ketamine (75 mg/kg) and xylazine (10 mg/kg) administered intraperitoneally.

Additional blood samples were collected from the abdominal aorta, centrifuged to obtain serum, and stored at −20 °C for subsequent analyses, including lipid profile determination and enzyme-linked immunosorbent assay (ELISA) for adiponectin. Ovarian, mesenteric, perirenal, and retroperitoneal adipose tissues were individually collected, carefully separated from surrounding tissues to avoid contamination with non-adipose tissues, and immediately weighed using a precision analytical balance. The weight of each adipose tissue depot was expressed relative to the final body weight of the animal.

### 2.6. Lipid Profile Assessment

Serum concentrations of total cholesterol, high-density lipoprotein (HDL), low-density lipoprotein (LDL), and triglycerides were determined using enzymatic colorimetric methods with commercial kits (Labtest Diagnóstica^®^, Lagoa Santa, MG, Brazil; 202202 IDO01). All assays were performed according to the manufacturer’s instructions regarding reagent preparation and calibration standards. Readings were obtained using a microplate reader (Thermo Scientific Multiskan FC^®^, Waltham, MA, USA).

### 2.7. Myocardial Contractility Assessment

Experiments using isolated left ventricular (LV) papillary muscles under isometric contraction conditions were performed as previously described [24]. After anesthesia, hearts were rapidly excised and immediately immersed in Krebs–Henseleit buffer containing (in mM): 135 NaCl, 4.6 KCl, 1.25 CaCl_2_, 1.15 MgSO_4_, 1.2 KH_2_PO_4_, and 5.5 glucose.

The right ventricular wall was removed to expose the interventricular septum, which was longitudinally sectioned to access the left ventricular papillary muscles. The posterior papillary muscle was dissected, attached to stainless-steel rings, and transferred to a chamber containing 20 mL of Krebs–Henseleit solution maintained at 29 °C and continuously aerated with a carbogenic mixture containing 95% O_2_ and 5% CO_2_, maintaining pH at 7.4. Preparations were connected to an isometric force transducer (TSD125, Biopac Systems Inc., Goleta, CA, USA) coupled to the MP100 system for developed force recording. Electrical stimulation was applied using isolated pulses (10–15 V; 5 ms) at 0.5 Hz through platinum electrodes. Muscles were stretched to the length associated with maximal force development (Lmax), and recordings began after a 60 min stabilization period. Contractile force was expressed as g/mg and normalized to papillary muscle weight.

During stabilization, contraction force, time to peak tension (TTP), and time to 90% relaxation (TR90%) were recorded. Subsequently, the following experimental protocols were performed in independent sets of preparations:

To evaluate isometric contraction force and temporal force parameters, the following baseline parameters were measured: contraction force, maximal positive and negative force derivatives (dF/dT), activation time (AT), and time to 90% relaxation (TR90%).

Sarcoplasmic reticulum activity was assessed using the post-pause potentiation (PPP) maneuver, which consists of temporarily interrupting electrical stimulation and analyzing the contraction immediately after stimulation resumption. Pauses of 15, 30, and 60 s were applied to investigate the capacity for Ca^2+^ storage and release by the sarcoplasmic reticulum. Force developed after each pause was normalized to the pre-pause contraction, allowing standardized comparisons between groups [25].

The effects of different extracellular Ca^2+^ concentrations (0.62, 1.25, and 2.5 mM) and the β-adrenergic agonist isoproterenol (10^−8^ to 10^−2^ M) on papillary muscle contraction force were also evaluated before and after experimental exposure.

Transsarcolemmal Ca^2+^ influx was assessed using the post-rest contraction (PRC) maneuver. Papillary muscles were incubated in Ca^2+^-free Krebs–Henseleit solution supplemented with caffeine (10 mM) to deplete intracellular Ca^2+^ stores through ryanodine receptor activation. Muscles remained at rest for 10 min without electrical stimulation. During the final two minutes, the perfusion solution was replaced with Krebs–Henseleit solution containing 1.25 mM Ca^2+^. The first contraction after electrical stimulation resumption predominantly reflects Ca^2+^ influx through the sarcolemma [26].

Tetanic contractions were used to assess myocardial contractile response under functional inactivation of the sarcoplasmic reticulum. Papillary muscle preparations were incubated for 30 min in Krebs–Henseleit solution containing caffeine (5 mM). Electrical stimulation was applied at 10 Hz for 15 s, and peak force and force plateau developed during stimulation were analyzed.

### 2.8. RNA Extraction and Real-Time PCR

Total RNA was extracted from cardiac tissue using TRIzol^®^ reagent (Invitrogen Life Technologies, Waltham, MA, USA). RNA purity and concentration were determined by absorbance ratios at 260/280 and 260/230 nm using a NanoDrop™ spectrophotometer (Thermo Scientific, Waltham, MA, USA). Complementary DNA (cDNA) synthesis was performed using the SuperScript™ III kit (Thermo Scientific). Gene expression was analyzed by quantitative real-time polymerase chain reaction (qPCR) using the QuantStudio™ system (Thermo Scientific) and SYBR Green fluorescent dye, as previously described [27]. Data were analyzed using the 2^−ΔΔCT^ method, and results were expressed as the ratio between target gene expression and the reference gene (β-actin).

The evaluated genes included SERCA2a (a sarcoplasmic reticulum protein in cardiomyocytes involved in Ca^2+^ reuptake), NCX1 (the sarcolemmal Na^+^/Ca^2+^ exchanger with functional expression in cardiomyocytes), PLB (a regulatory protein of the cardiac sarcoplasmic reticulum that modulates SERCA2a activity), TNF-α (a pro-inflammatory cytokine with paracrine and autocrine effects produced by immune cells and also by the myocardium under pathological conditions), leptin (an adipokine derived from adipose tissue with systemic cardiovascular actions and potential direct effects on cardiac tissue), and MCP-1 (a chemokine expressed in endothelium, adipose tissue, and immune cells involved in inflammatory signaling and monocyte recruitment in the myocardium). Primer sequences were as follows: SERCA2a (forward: TCGAAGAAGGTGAAGAAACGA; reverse: CTTGCCCATTTCAGGTTCAT), NCX1 (forward: CACCTGTGGAGAGCTGGAAT; reverse: AGACGGGGTTCTCCAATCTC), PLB (forward: CGTCAGAACCTCCAGAACCT; reverse: ATCGTGACCCTTCACGACGA), TNF-α (forward: AAATGGGCTCCCTCTCATCAGTTC; reverse: TCTGCTTGGTGGTTTGCTACGAC), and leptin (forward: ATTTCACACACGCAGTCGGT; reverse: CCAGGGTCTGGTCCATCTTG); MCP-1 (forward: TGTCTCAGCCAGATGCAGTT, reverse: CAGCCGACTCATTGGGATCA.

### 2.9. Enzyme-Linked Immunosorbent Assay (ELISA)

Plasma adiponectin concentrations were determined using ELISA. Commercial rat ELISA kits were used for quantification (Rat Adiponectin/Acrp30 DuoSet ELISA Features; R&D Systems, Minneapolis, MN, USA; catalog number DY3100-05). Assays were performed according to the manufacturer’s instructions, and absorbance was measured at 450 nm using a Thermo Scientific Multiskan™ FC microplate reader (Thermo Fisher Scientific, Waltham, MA, USA). Analyses followed protocols previously described in the literature [28,29].

### 2.10. Statistical Analysis

Data are expressed as mean ± standard error of the mean (SEM). Comparisons between experimental groups were performed using Student’s *t*-test. Analyses involving two factors were performed using two-way analysis of variance (two-way ANOVA), followed by Bonferroni’s post hoc test. Values of *p* < 0.05 were considered statistically significant. All statistical analyses and graph construction were performed using GraphPad Prism 8.0 software (GraphPad Software Inc., San Diego, CA, USA).

## 3. Results

### 3.1. General Characteristics

The high-refined carbohydrate diet administered for 15 days did not alter final body mass, despite reductions in chow intake (7.75%) and water consumption (18.24%), with no impact on energy intake or feed efficiency. The HCD group exhibited an increase in ovarian adipose tissue mass (36.98%), without changes in the other fat depots. Left ventricular papillary muscle mass did not differ between groups. Regarding the metabolic profile, no changes were observed in fasting glucose or total cholesterol levels; however, increased triglyceride levels (24.56%) and reduced HDL (19.66%) and LDL (16.4%) concentrations were detected. These data are presented in Table 2.

### 3.2. Glucose Metabolism

**Figure 1:**
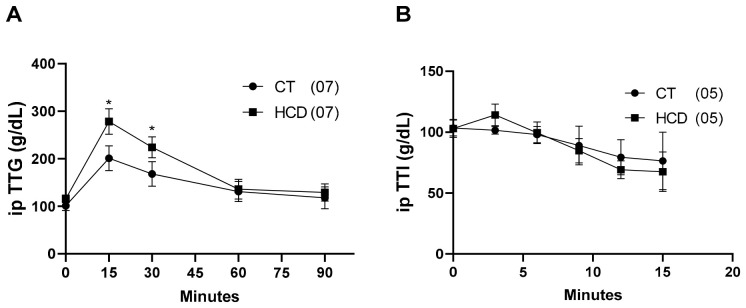
Evaluation of glucose metabolism. Glucose tolerance test (GTT) (**A**) and insulin tolerance test (ITT) (**B**) in Wistar rats treated for 15 days with a control diet (CT) or a high refined carbohydrate diet (HCD). Data are expressed as mean ± SEM. * *p* < 0.05 vs. CT; Statistical analysis was performed using Student’s *t*-test. The number of animals per group is indicated in parentheses.

In the GTT, the HCD group exhibited higher glycemic values at 15 min (38.40%) and 30 min (33.19%) compared with the control group, returning to baseline levels after 60 min (3.81%). In the ITT, no differences were observed between groups. These results are presented in Figure 1.

### 3.3. Inflammatory Parameters

**Figure 2:**
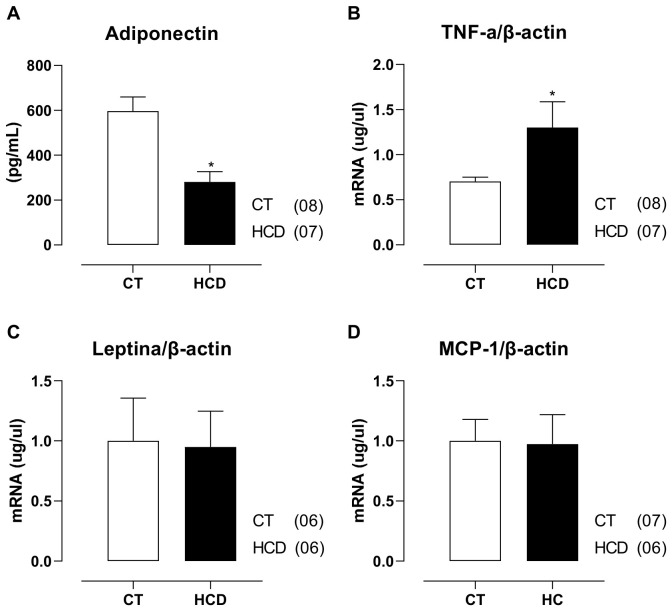
Evaluation of serum levels of adipokines and inflammatory cytokines in Wistar rats from the control (CT) and high-carbohydrate diet (HCD) groups after 15 days of exposure. (**A**) Adiponectin. (**B**) TNF-α. (**C**) Leptin. (**D**) MCP-1. Numbers in parentheses indicate the number of animals analyzed in each group. Data are expressed as mean ± standard error of the mean (SEM). Student’s *t*-test. * *p* < 0.05 vs. CT.

In the evaluation of inflammatory and metabolic mediators, the HCD group exhibited reduced serum adiponectin levels (Figure 2A). Additionally, gene expression analysis in cardiac tissue revealed increased TNF-α expression compared with the CT group (Figure 2B), whereas the expression levels of leptin and MCP-1 remained unchanged (Figure 2C,D). This profile suggests the early establishment of a metabolic-inflammatory dysregulation state, characterized by the loss of mediators with cardioprotective properties and the concomitant increase in pro-inflammatory cytokines, even under short-term dietary exposure.

### 3.4. Myocardial Contractility

**Figure 3:**
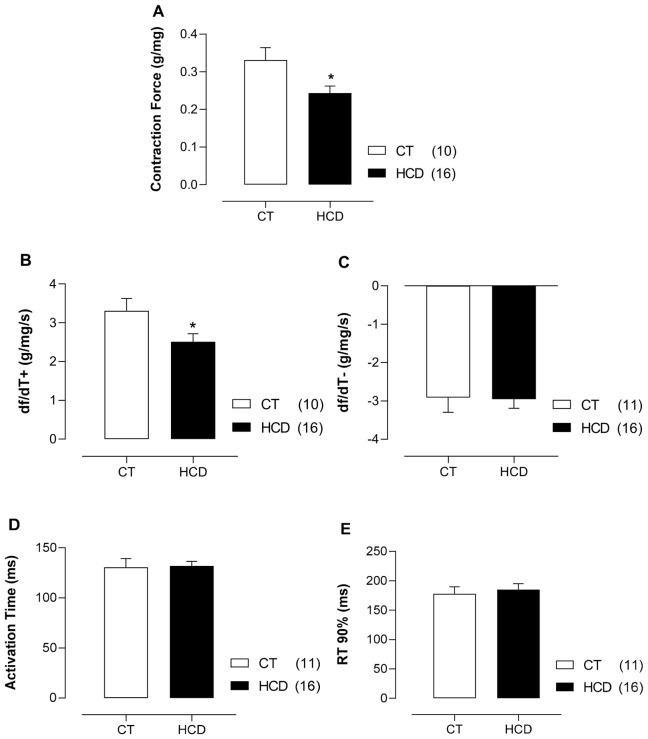
Contractile force and contractility parameters of left ventricular papillary muscles from Wistar rats in the control (CT) and high-carbohydrate diet (HCD) groups after 15 days of exposure. (**A**) Isometric contractile force; (**B**) maximum positive force derivative (dF/dt^+^); (**C**) maximum negative force derivative (dF/dt^−^); (**D**) contraction activation time; (**E**) time to 90% relaxation (RT90%). Numbers in parentheses indicate the number of papillary muscles studied. Values are expressed as mean ± SEM. Student’s *t*-test. * *p* < 0.05 vs. CT.

Exposure to the high-refined carbohydrate diet for 15 days reduced developed force in the HCD group compared with the CT group (Figure 3A). This reduction was accompanied by a significant decrease in dF/dt^+^, indicating impairment in the rate of force development during contraction (Figure 3B), despite the absence of differences in activation time (Figure 3D). Conversely, no differences were observed in dF/dt^−^ (Figure 3C) or in time to TR90% (Figure 3E) between groups, suggesting preservation of myocardial relaxation temporal parameters.

### 3.5. Modulation of Myocardial Contractility and Ca^2+^ Handling

**Figure 4:**
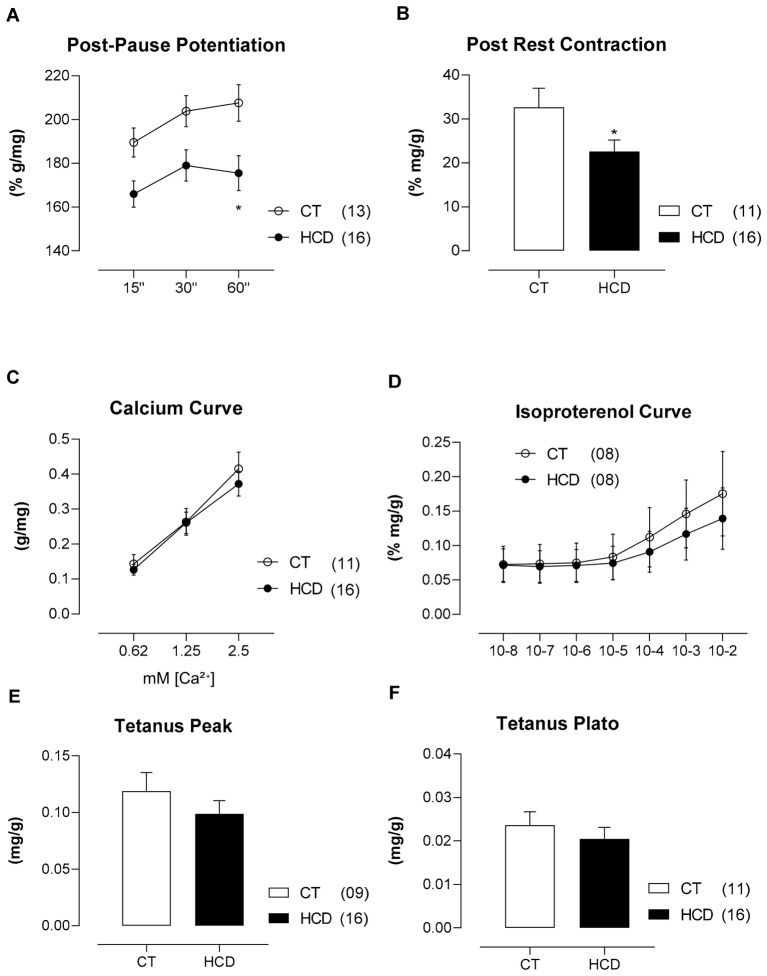
Functional assessment of excitation–contraction coupling in left ventricular papillary muscles from Wistar rats in the control (CT) and high-carbohydrate diet (HCD) groups after 15 days of exposure. (**A**) Post-pause potentiation (PPP). (**B**) Post-rest contraction (PRC). (**C**) Extracellular calcium response curve. (**D**) Response to β-adrenergic stimulation (Isoproterenol). (**E**) Peak tetanic contraction. (**F**) Tetanic contraction plateau. Numbers in parentheses indicate the number of samples per group. Data are expressed as mean ± standard error of the mean (SEM). Comparisons between two groups were performed using Student’s *t*-test. For two-factor analyses, two-way analysis of variance (ANOVA) followed by Bonferroni’s post hoc test was used. * *p* < 0.05 vs. CT.

Given the reduction in contractile force and dF/dt^+^ in the HCD group, the post-rest potentiation (PRP) protocol was applied to evaluate the participation of the SR in intracellular Ca^2+^ handling. A reduction in contractile response after a 60 s pause was observed (Figure 4A), suggesting impairment in the SR capacity for Ca^2+^ accumulation and release. Consistently, the HCD group exhibited lower PRC force (Figure 4B), indicating a possible reduction in extracellular calcium influx and, consequently, lower Ca^2+^ availability for the contractile process.

In contrast, the response to increased extracellular Ca^2+^ concentration and β-adrenergic stimulation with isoproterenol did not differ between groups (Figure 4C,D). Additionally, tetanic contractions showed similar peak and plateau values in CT and HCD groups (Figure 4E,F). Taken together, these findings indicate that although intracellular Ca^2+^ influx and handling were impaired, myofibrillar responsiveness and the capacity to sustain contractions remained preserved.

### 3.6. Expression of Genes Involved in Myocardial Contractility

**Figure 5:**
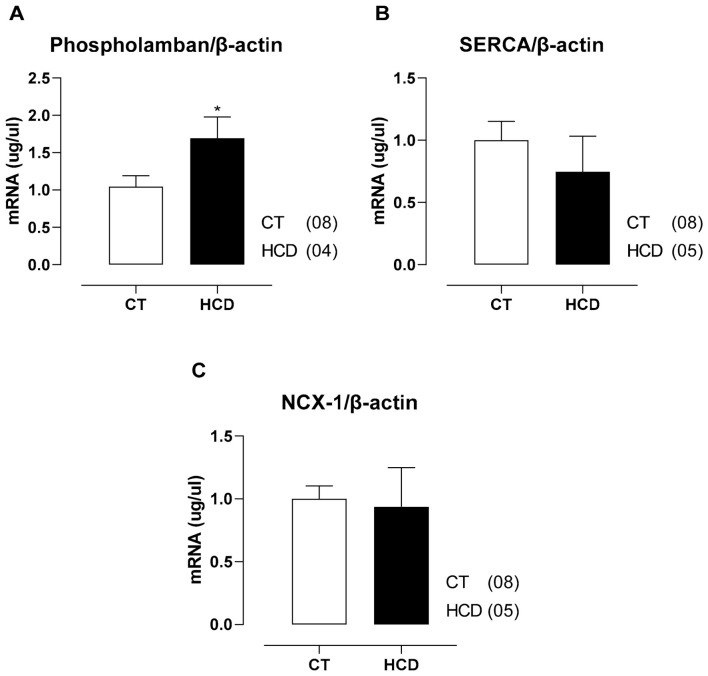
Evaluation of the gene expression of proteins involved in intracellular Ca^2+^ handling in Wistar rats from the control (CT) and high-carbohydrate diet (HCD) groups after 15 days of exposure. (**A**) Phospholamban (PLB); (**B**) SERCA; (**C**) NCX-1. Numbers in parentheses indicate the number of papillary muscles analyzed. Values are expressed as mean ± SEM. Student’s *t*-test. * *p* < 0.05 vs. CT.

At the molecular level, the analysis of components involved in intracellular Ca^2+^ handling revealed increased PLB expression in the HCD group, without alterations in SERCA2a and NCX-1 expression (Figure 5A–C). Considering the regulatory role of PLB on SERCA2a, its increased expression suggests negative modulation of this pump activity, potentially impairing Ca^2+^ reuptake into the sarcoplasmic reticulum. This imbalance among regulatory proteins involved in Ca^2+^ cycling is consistent with a scenario of early subcellular dysfunction, in which alterations in SR regulation precede structural modifications or broader changes in the contractile machinery.

## 4. Discussion

The present study demonstrated for the first time that short-term exposure to a high-refined carbohydrate diet is sufficient to induce early metabolic, inflammatory, and cardiac functional alterations in female Wistar rats, even in the absence of obesity. The main findings include increased ovarian adipose tissue, glucose intolerance, hypertriglyceridemia, reduced HDL levels, decreased circulating adiponectin, elevated TNF-α levels, and impaired myocardial contractility, characterized by reduced developed force and dF/dt^+^. In addition, functional analyses revealed alterations in intracellular Ca^2+^ influx and handling, evidenced by reduced post-rest potentiation and post-rest contraction responses. Notably, these alterations occurred with preserved β-adrenergic responsiveness and myofibrillar sensitivity, suggesting early impairment of sarcoplasmic reticulum function.

Despite the absence of a significant increase in body mass, consumption of the high-refined carbohydrate diet induced early metabolic alterations characterized by increased ovarian adipose tissue, glucose intolerance, hypertriglyceridemia, and reduced HDL levels. These findings suggest that diet quality plays a more relevant role than caloric excess in the induction of early cardiometabolic alterations, a pattern also observed in males subjected to the same experimental protocol [14]. The high glycemic index of the diet may favor episodes of transient hyperinsulinemia and stimulate de novo lipogenesis, contributing to visceral fat accumulation [30].

It is important to emphasize that the modified diet used in this study differed from the control diet not only due to its higher content of refined carbohydrates, but also due to an increased lipid content and a relative reduction in the proportion of proteins. Part of the lipids originated from the condensed milk, whose composition is characterized by milk fat rich in saturated fatty acids, which have been associated with alterations in lipid metabolism and inflammatory pathways when consumed in excess [31]. In addition, the lower relative proportion of proteins in the diet may influence processes related to energy metabolism and the maintenance of metabolic homeostasis [32]. However, considering that the main nutritional modification consisted of a marked increase in the intake of refined carbohydrates and simple sugars, and that no signs of protein malnutrition or weight loss were observed, it is believed that the observed changes predominantly reflect the effects of the overall dietary pattern, making it impossible to fully dissociate the individual contribution of each macronutrient [33,34].

The increase in ovarian adipose tissue observed in the HCD group suggests early expansion of visceral adiposity, a metabolically active condition associated with greater secretion of pro-inflammatory adipokines and cytokines involved in cardiovascular dysfunction [35]. In experimental models of high-refined carbohydrate diets, increased visceral fat has been associated with systemic inflammation, metabolic resistance, and increased cardiometabolic risk [11,12]. In parallel, glucose intolerance, hypertriglyceridemia, and reduced HDL levels represent classical alterations associated with the development of atherosclerosis, endothelial dysfunction, and impairment of cardiovascular homeostasis [36,37]. Taken together, these results characterize a metabolically unhealthy phenotype, even in the absence of obesity.

Considering the findings discussed above, important cytokines involved in cardiometabolic regulation were evaluated. Adiponectin levels were reduced in the HCD group, which is associated with the early loss of cardioprotective mechanisms involved in the regulation of energy metabolism and myocardial homeostasis [38,39], since adiponectin acts through activation of the AMPK pathway, favoring the maintenance of mitochondrial function and intracellular Ca^2+^ handling [40]. In contrast, the increase in TNF-α indicates the establishment of a low-grade inflammatory state, potentially capable of interfering with excitation–contraction coupling and the function of sarcoplasmic reticulum regulatory proteins [41]. In this context, the imbalance between anti-inflammatory and pro-inflammatory mediators may represent an early trigger for myocardial dysfunction induced by the high-refined carbohydrate diet [42]. Additionally, the absence of alterations in leptin and MCP-1 suggests an early stage of metabolic and inflammatory dysfunction [43,44], characterized by a still restricted profile of alterations, in which adiponectin and TNF-α stand out among the evaluated mediators.

The functional findings demonstrated reduced developed force and dF/dt^+^ in the HCD group, indicating early impairment of the myocardial contractile activation phase. In contrast, preservation of dF/dt^−^ and relaxation-related parameters suggests that the observed dysfunction occurs selectively in contractile function, possibly associated with early alterations in intracellular Ca^2+^ cycling. The present study demonstrates that contractile alterations may arise during the initial phases of exposure to a high-refined carbohydrate diet, even in the absence of significant weight gain. This finding suggests high myocardial sensitivity to diet-induced metabolic and inflammatory alterations, reinforcing the potential early cardiovascular impact of this dietary pattern.

To investigate the mechanisms involved in the observed contractile dysfunction, post-rest potentiation PRP and post-rest contraction PRC protocols were employed, both widely used for the functional evaluation of Ca^2+^ storage and release by the sarcoplasmic reticulum SR and sarcolemmal calcium influx. Reduced contractile responses after PRP and PRC in the HCD group indicate impairment of intracellular Ca^2+^ dynamics, particularly related to reduced SR capacity for Ca^2+^ accumulation and mobilization, as well as reduced sarcolemmal influx. In contrast, preservation of responses to extracellular Ca^2+^, β-adrenergic stimulation, and tetanic contractions indicates maintenance of β-adrenergic responsiveness and myofibrillar sensitivity. These findings support the hypothesis of reduced Ca^2+^ availability rather than a generalized failure of excitation–contraction coupling.

Experimental evidence demonstrates that adiponectin exerts a cardioprotective role in calcium homeostasis by modulating proteins related to the SR and favoring excitation–contraction coupling efficiency [45]. Among the main regulators of this process are the SERCA2a, responsible for Ca^2+^ reuptake into the SR during diastole, and PLB, a regulatory protein that, in its dephosphorylated form, inhibits SERCA2a activity [46]. Therefore, alterations in the expression or functional activity of these proteins may impair Ca^2+^ reuptake and storage in the SR, reducing the amount available for release during myocardial contraction and contributing to the observed contractile dysfunction.

Although no alterations were observed in SERCA2a and NCX-1 expression, increased PLB expression was detected in the HCD group. Considering that PLB exerts an inhibitory effect on SERCA2a in its dephosphorylated form, increased PLB expression may be associated with reduced pump activity, depending on its phosphorylation state, which may impair Ca^2+^ reuptake by the SR and limit the Ca^2+^ content available for release during contraction [47,48]. This condition may lead to cardiomyocyte energetic dysfunction, with consequent limitation of ATP availability for proper cellular functioning [39,40], thereby impairing Ca^2+^ handling [42].

Inflammation may favor increased PLB expression through the activation of intracellular signaling pathways triggered by pro-inflammatory cytokines [17,49]. Among these cytokines, TNF-α plays a central role by binding to specific receptors on the cardiomyocyte membrane and promoting activation of intracellular cascades, especially the NF-κB pathway, thereby modulating the expression of genes related to cardiac remodeling and proteins involved in intracellular Ca^2+^ handling, favoring adaptive alterations in the SR [50].

Tang and colleagues (2025) also demonstrated that inflammatory mediators, such as MIF, can modulate cardiac function through PBL phosphorylation [51]. Although that study evaluated mechanisms involving PLB phosphorylation, the findings of the present study demonstrated increased gene expression of this protein, suggesting that the inflammatory process may also act at the transcriptional level, regulating its expression. Therefore, inflammation may represent an important mechanism associated with myocardial molecular alterations, contributing to adaptations or dysfunctions in cardiac calcium regulation and, consequently, to alterations in cardiac contractility.

Taken together, the findings of the present study allow the proposal of a model in which consumption of a high-refined carbohydrate diet induces early metabolic alterations associated with low-grade inflammation, characterized by reduced adiponectin and increased TNF-α levels. This metabolic-inflammatory environment may favor increased PLB expression and consequent functional inhibition of SERCA2a, impairing Ca^2+^ reuptake by the SR and reducing intracellular Ca^2+^ content available for myocardial contraction. Some limitations should be considered, including the absence of protein evaluation and assessment of PLB phosphorylation status, as well as the lack of direct measurement of SERCA2a activity and echocardiographic assessment of cardiac function. Nevertheless, the study consistently demonstrates early functional alterations associated with refined carbohydrate consumption.

Future studies may investigate the effects of prolonged dietary exposure on cardiac remodeling, mitochondrial function, oxidative stress, and alterations in proteins involved in intracellular Ca^2+^ handling. In this regard, studies with longer intervention periods are necessary to evaluate the progression of these early alterations and to determine whether maintenance of the metabolic stimulus leads to impairment of other mechanisms involved in myocardial contractility, particularly those related to Ca^2+^ handling and the activity of proteins responsible for its extrusion, as well as how these changes may impact cardiac structural remodeling. Furthermore, considering sex-related differences in cardiovascular and metabolic responses, additional studies using male animals subjected to the same experimental diet are still necessary in order to compare potential sex-related pathophysiological differences.

## 5. Conclusions

The present study demonstrates that short-term exposure to a high-refined carbohydrate diet is sufficient to induce early metabolic, inflammatory, and cardiac functional alterations in female Wistar rats, even in the absence of obesity. These alterations were characterized by increased ovarian adipose tissue, glucose intolerance, dyslipidemia, reduced adiponectin levels, increased TNF-α levels, and impaired myocardial contractility. Additionally, the functional findings indicate selective impairment in intracellular Ca^2+^ influx and handling, as evidenced by the reduction in post-rest potentiation and post-rest contraction responses, whereas transsarcolemmal Ca^2+^ influx, β-adrenergic responsiveness, and myofibrillar sensitivity remained preserved.

Taken together, these data indicate that diet-induced metabolic alterations are associated with early cardiac functional modifications, even in the absence of obesity or detectable structural alterations. These findings suggest the involvement of the SR and the inflammatory-metabolic axis as relevant components in the cardiac alterations observed in this experimental model. A schematic summary of the main experimental findings is presented in Graphical Abstract.

## Figures and Tables

**Table 1:** Comparison of energy density and macronutrient distribution between the groups CT and HCD.

Type of Diet	Standard Feed (Socil^®^)	High-Refined Carbohydrate Diet (HCD)
Energy density	4.0 kcal/g	4.4 kcal/g
Carbohydrates	65.80%	74.20%
Lipids	3.10%	5.80%
Proteins	31.10%	20.00%

Energy density and percentage distribution of carbohydrates, lipids, and proteins in the standard diet (Socil^®^ chow) and the high-carbohydrate diet (HCD) used in the study. Values were calculated based on the nutritional composition provided by the manufacturers of the ingredients used in the diet formulation. Source: prepared by the authors.

**Table 2:** General characteristics.

Variables	CT	HCD	*p*-Value
**Nutritional Status**			
Initial body weight (g)	219.03 ± 3.13 (28)	219.64 ± 2.42 (28)	0.8788
Final body weight (g)	240.42 ± 2.92 (28)	244.21 ± 3.20 (28)	0.3866
Weight gain (g)	21.39 ± 1.52 (28)	24.57 ± 1.54 (28)	0.1484
Food intake (g/dia)	17.54 ± 0.43 (28)	16.18 ± 0.44 * (28)	0.0349
Water intake (mL/dia)	25.93 ± 0.89 (28)	21.2 ± 0.59 * (28)	0.0001
Energy efficiency (Kcal)	0.34 ± 0.01 (28)	0.33 ± 0.01 (28)	0.6335
Energy consumption (Kcal)	70.17 ± 1.75 (28)	70.69 ± 1.90 (28)	0.8419
**Adipose Tissue**			
Ovarian (g)	3.11 ± 0.29 (14)	4.26 ± 0.40 * (14)	0.0297
Mesenteric (g)	1.46 ± 0.07 (14)	1.81 ± 0.16 (14)	0.0738
Perirenal l (g)	0.73 ± 0.11 (14)	0.96 ± 0.10 (14)	0.1303
Retroperitoneal l (g)	1.66 ± 0.12 (14)	1.90 ± 0.20 (14)	0.3343
**Biocheminal Parameters**			
Glucose (mg/dL)	103.89 ± 13.12 (28)	99.96 ± 11.75 (28)	0.2433
LDL (mg/dL)	63.39 ± 4.07 (9)	53.01 ± 0.46 * (9)	0.0222
HDL (mg/dL)	51.16 ± 3.41 (9)	41.10 ± 2.98 * (9)	0.0414
Total cholesterol (mg/dL)	132.53 ± 9.02 (9)	121.91 ± 26.25 (9)	0.7071
Triglyceride (mg/dL)	90.10 ± 4.62 (9)	112.23 ± 6.54 * (9)	0.0139
**Anatomical Analysis**			
Weight of papillary (g)	6.61 ± 0.54 (17)	5.59 ± 0.45 (20)	0.1562

The data are presented as mean ± standard error of the mean (SEM), numbers in parentheses indicate the number of samples per group. * *p* < 0.05 indicates a statistically significant difference between groups (Student *t*-test).

## Data Availability

The original contributions presented in this study are included in the article. Further inquiries can be directed to the corresponding author.

## Abbreviations

The following abbreviations are used in this manuscript:

AT	Activation Time
cDNA	Complementary DNA
CT	Control Group
df/dt	Maximal Positive and Negative Force Derivatives
ELISA	Enzyme-Linked Immunosorbent Assay
GTT	Glucose Tolerance Testing
HCD	High-Carbohydrate Diet
HDL	High-Density Lipoprotein
ip	Intraperitoneally
ITT	Insulin Tolerance Testing
LDL	Low-Density Lipoprotein
Lmax	Maximal Force Development
LV	Left Ventricular
PBL	Phospholamban
PPP	Post-Pause Potentiation
PRC	Post-Rest Contraction
PRP	Post-Rest Potentiation
qPCR	Quantitative Real-Time Polymerase Chain Reaction
SR	Sarcoplasmic Reticulum
TNF-α	Tumor Necrosis Factor Alpha
TTP	Time to Peak Tension
TR90%	Time to 90% Relaxation
WHO	World Health Organization
